# Microbial Communities and Physicochemical Characteristics of Traditional Dajiang and Sufu in North China Revealed by High-Throughput Sequencing of 16S rRNA

**DOI:** 10.3389/fmicb.2021.665243

**Published:** 2021-08-30

**Authors:** Tingting Liang, Xinqiang Xie, Jun Ma, Lei Wu, Yu Xi, Hui Zhao, Longyan Li, Haixin Li, Ying Feng, Liang Xue, Moutong Chen, Xuefeng Chen, Jumei Zhang, Yu Ding, Qingping Wu

**Affiliations:** ^1^School of Food and Biological Engineering, Shaanxi University of Science and Technology, Xi’an, China; ^2^Guangdong Provincial Key Laboratory of Microbial Safety and Health, State Key Laboratory of Applied Microbiology Southern China, Institute of Microbiology, Guangdong Academy of Sciences, Guangzhou, China; ^3^Department of Food Science & Technology, Institute of Food Safety and Nutrition, Jinan University, Guangzhou, China

**Keywords:** Dajiang, Sufu, physicochemical index, bacterial community, functional analysis

## Abstract

The process of soybean fermentation has been practiced for more than 3,000 years. Although Dajiang and Sufu are two popular fermented soybean products consumed in North China, limited information is available regarding their microbial composition. Hence, the current study sought to investigate, and compare, the physicochemical indicators and microbial communities of traditional Dajiang and Sufu. Results showed that the titratable acidity (TA), and salinity, as well as the lactic acid, and malic acid contents were significantly higher in Sufu samples compared to Dajiang. Furthermore, Sufu samples contain abundant sucrose and fructose, while the acetic acid content was lower in Sufu compared to Dajiang samples. Moreover, the predominant bacterial phyla in Dajiang and Sufu samples were Firmicutes and Proteobacteria, while the major genera comprise *Bacillus*, *Lactobacillus*, *Tetragenococcus*, and *Weissella*. Moreover, Dajiang samples also contained abundant *Pseudomonas*, and *Brevundimonas* spp., while *Halomonas*, *Staphylococcus*, *Lysinibacillus*, *Enterobacter*, *Streptococcus*, *Acinetobacter*, and *Halanaerobium* spp. were abundant in Sufu samples. At the species level, *Bacillus velezensis*, *Tetragenococcus halophilus*, *Lactobacillus rennini*, *Weissella cibaria*, *Weissella viridescens*, *Pseudomonas brenneri*, and *Lactobacillus acidipiscis* represented the major species in Dajiang, while *Halomonas* sp., *Staphylococcus equorum*, and *Halanaerobium praevalens* were the predominant species in Sufu. Acetic acid and sucrose were found to be the primary major physicochemical factor influencing the bacterial communities in Dajiang and Sufu, respectively. Furthermore, *Bacillus subtilis* is strongly correlated with lactic acid levels, *L. acidipiscis* is positively correlated with acetic acid levels, while *Staphylococcus sciuri* and *S. equorum* are strongly, and positively, correlated with malic acid. Following analysis of carbohydrate and amino acid metabolism in all samples, cysteine and methionine metabolism, as well as fatty acid biosynthesis-related genes are upregulated in Dajiang compared to Sufu samples. However, such as the *Staphylococcus*, *W. viridescens*, and *P. brenneri*, as potentially foodborne pathogens, existed in Dajang and Sufu samples. Cumulatively, these results suggested that Dajiang and Sufu have unique bacterial communities that influence their specific characteristics. Hence, the current study provides insights into the microbial community composition in Dajiang and Sufu samples, which may facilitate the isolation of functional bacterial species suitable for Dajiang and Sufu production, thus improving their production efficiency.

## Introduction

Fermented soybean products are widely consumed in countries across the world, particularly in Eastern Asia, including China, where Dajiang (An et al., 2019), Sufu (Wan et al., 2020), douchi, and soybean sauce (Feng et al., 2014) are popular, while natto is common in Japan (Gao et al., 2018), ganjang (Cho and Seo, 2007), doenjang (Kim et al., 2009) and cheonggukjang (Nam et al., 2012) in Korea, and thua nao in Thailand (Inatsu et al., 2010). Among these products, Dajiang (“soybean paste”) and Sufu (“Chinese cheese”) are popular traditional food products in northeast China (Xie et al., 2018). Dajiang is a flavouring ingredient that is produced from soybeans using naturally inoculated microorganisms under certain temperatures and humidity (An et al., 2019); it has been described as having numerous health benefits, including reducing blood pressure, decreasing cholesterol levels, lowering blood glucose, and improving intestinal permeability (Kim et al., 2016). Sufu is another well-known fermented soybean in northeast China that is commonly used as the starting materials for tofu (soybean curd) (Wan et al., 2020). Sufu is also rich in nutrients, taste, and flavour, with a fine texture (Li et al., 2010). Although both Dajiang and Sufu are traditional food products that are commonly consumed in North China, cases of associated food poisoning have been reported and, thus, it is necessary to stablish measures to ensure quality and safety in Dajiang and Sufu production. Additionally, Dajiang and Sufu have unique flavours appearances, and characteristics (e.g., pH, acidity, salinity, and odour) that may result from differences in their manufacturing processes or bacterial composition.

Like other fermented foods, such as Suan-cai (Xiao et al., 2020), cheese, yoghurt, and vinegar, the bacterial community plays an important role in Dajiang and Sufu fermentation. In fact, the bacterial community composition reportedly has a major influence on the taste, flavour, texture, colour, and nutritional properties of the resulting fermented foods (Marshall and Tamime, 1997). Specifically, Cao et al. (2017) reported that *Lactobacillus sakei*, *Pediococcus acidilactici*, and *Weissella thailandensis* significantly impact the quality of fermented sausage, while their metabolites provide a flavour profile. Additionally, bacteria from the genera *Bacillus* and *Lactobacteria* predominate in bean sauce mash (Lee et al., 2010); while *Staphylococcus*, *Bacillus*, and *Leuconostoc* are key microbiota responsible for soybean fermentation (Sun et al., 2018). However, the dominant microbiota differs greatly in different traditional fermented soybean products, due to the differences in raw materials, geography, climate, as well as the fermentation methods. For example, Rui et al. (2017) suggested that doubanjiang-meju predominantly includes the genera *Tetragenococcus*, *Lactobacillus*, *Staphylococcus*, *Acinetobacter*, *Pseudomonas*, and *Streptococcus*; whereas, in traditional Baixi sufu the dominant bacteria are *Enterococcus*, *Lactococcus*, and *Bacillus* spp. (Wan et al., 2020). Still further, Kim et al. (2010) reported that *Bacillus* spp. Are the predominate species in traditional Chinese fermented soybean pastes, while Tetragenococcus halophilus predominates in Japanese samples.

In general, Dajiang is homemade, naturally inoculated with microbiota, and requires 3 or 4 months for production (Xie et al., 2019). However, the fermentation process of both traditional Dajiang and Sufu may introduce pathogenic bacteria from the environment. Hence, when optimising the production efficiency and safety of Dajiang and Sufu, it is crucial to ensure appropriate measures are taken to prevent the colonisation of traditional fermented soybeans with pathogenic bacteria or other bacterial contaminants (Sun et al., 2015). Therefore, to ensure that the required bacteria are present, while preventing bacterial contamination, it is necessary to gain a more comprehensive understanding regarding the microbial composition of Dajiang and Sufu, while also characterising the factors responsible for their unique flavours and characteristics.

The current study, therefore, sought to evaluate, and compare, the physicochemical characteristics, bacterial communities, and biological functions of Dajiang and Sufu products collected from North China, an area with continental monsoon climate. The relationship between bacterial diversity and physiochemical indices was also investigated. Hence, the primary goal of this study was to identify differences in the microbial community composition and functional profiles of Dajiang and Sufu to guide the selection of optimal and appropriate strains for the industrial production of Dajiang and Sufu in North China, while simultaneously improving the quality and safety of traditional Chinese fermented soybean products.

## Materials and Methods

### Sample Collection

All 19 fermented soybean samples were collected from North China in August 2019. Most samples were collected from farmer households and markets that fermented soybean samples for self-consumption. The samples comprised 14 Dajiang samples (DJ_143, DJ_146, DJ_50, DJ_145, DJ_49, DJ_246, DJ_247, DJ_227, DJ_241, DJ_240, DJ_391, DJ_393, DJ_400, and DJ_401), and five Sufu samples (SF_224, SF_245, SF_244, SF_394, and SF_249). Dajiang samples were prepared by soaking and steaming soybeans, which were then mixed with flour and allowed to spontaneously ferment room temperature. Yellow mycelium appeared after 3 days at which point the fermented soybeans and 16–18% salt water was added to a jar. The contents were stirred once or twice per day with sun exposure for 3–4 months, before eating. Sufu was prepared by cutting tofu into pieces and placing them in a bowl for approximately 20 days, at which point white mycelia had covered the surface of the bean curd. The tofu cubes were then placed in salt inside the rolling, with chilli powder added. It was then left to pickle at room temperature for approximately 10 days to allow spontaneous fermentation. All samples were placed into aseptic bags and sealed; they were then placed into a foam box filled with dry ice, and immediately transported to the laboratory where they were stored at appropriately −80°C until analysis.

### Physiochemical Analysis

The pH, titratable acidity (TA), and salinity of samples were measured according to previously described protocols (Wan et al., 2020), according to the National Standard method (SB/T10170, 2007). Glucose, sucrose, and fructose levels were determined using a high-performance liquid chromatography-differential refraction detector (HPLC-DRD), as described previously (Yanping, 2016). The concentrations of organic acids (lactic acid, malic acid, and acetic acid) were measured according to previously published protocols (Darji et al., 2013; Kuan et al., 2019), with slight modifications.

### DNA Extraction and Polymerase Chain Reaction Amplification

Microbial DNA from 19 fermented soybean samples was extracted using the E.Z.N.A.^®^ soil DNA Kit (Omega Bio-tek, United States), according to the manufacturer’s instructions. The concentration and purity of genomic DNA were determined using a NanoDrop 2000 UV-vis spectrophotometer (Thermo Fisher Scientific, United States), and DNA quality was assessed using 1% agarose gel electrophoresis. The V3–V4 hypervariable regions of bacterial 16S rRNA genes were amplified with primers 338F (5′-ACTCCTACGGGAGGCAGCAG-3′) and 806R (5′-GGACTACHVGGGTWTCTAAT-3′) using a thermocycler (GeneAmp 9700, ABI, United States). The polymerase chain reaction (PCR) conditions were as follows: 3 min of denaturation at 95°C, 27 cycles of 30 s denaturation at 95°C, 30 s of annealing at 55°C, and 45 s of elongation at 72°C, with a final 10 min extension at 72°C. The PCR mixture contained 4 μL of 5× FastPfu Buffer, 2 μL of 2.5 mM dNTPs, 0.8 μL of each primer (5 μM), 0.4 μL FastPfu Polymerase, and 10 ng template DNA. Amplicons were extracted from a 2% agarose gel and further purified using the AxyPrep DNA Gel Extraction Kit (Axygen Biosciences, United States) and quantified using QuantiFluor^TM^-ST (Promega, United States), according to the manufacturer’s instructions. All PCR amplifications were repeated three times.

### Illumina Miseq Sequencing

Purified amplicons were pooled in equimolar concentrations and were paired-end sequenced using an Illumina MiSeq PE300 platform/NovaSeq PE250 platform (Illumina, San Diego, CA, United States), according to the standard protocols by Majorbio Bio-Pharm Technology Co., Ltd. (Shanghai, China). The raw reads were deposited in the NCBI Sequence Read Archive database (Accession Number: PRJNA723724).

### Sequencing Data Processing

The raw 16S rRNA gene sequencing reads were demultiplexed, quality-filtered using fastp version 0.20.0 (Chen S. et al., 2018), and merged using FLASH version 1.2.7 (Mago and Salzberg, 2011) with the following criteria: (i) the 300 bp reads were truncated at any site receiving an average quality score <20 over a 50 bp sliding window, while truncated reads <50 bp were discarded. Reads containing ambiguous characters were also discarded. (ii) Only overlapping sequences longer than 10 bp were assembled according to their overlapped sequence. The maximum mismatch ratio of the overlap region was 0.2. Reads that could not be assembled were discarded. (iii) Samples were distinguished based on the barcode and primers, the sequence direction was adjusted, and the precise barcode was matched using a two-nucleotide mismatch in primer matching.

Operational taxonomic units (OTUs) with 97% similarity cut-off were clustered using UPARSE (version 7.1) (Edgar, 2013), and chimeric sequences were identified and removed. The taxonomy of each OTU representative sequence was analysed using RDP Classifier (Wang, 2007) against the 16S rRNA database (Silva v138) with a 0.7 confidence threshold.

### Statistical Analysis

The differences in physicochemical indicators among all groups were performed using Student’s *t*-test. A *P*-value <0.05 was considered significant. All data are described as the mean ± standard deviation. The graphs were generated in GraphPad Prism 7 (GraphPad Software, Inc., La Jolla, CA, United States). Rarefaction analysis and alpha diversities were performed using Mothur (version v.1.30.1)^1^. Bray Curtis similarity clustering analysis was performed using R package (R 3.0.2)^2^. Mann–Whitney U-test was used to assess the different taxonomies of the bacterial communities. Spearman correlation analysis was used to determine the relationship between the bacterial communities and physicochemical indicators (pH, TA, salinity, glucose, sucrose, fructose, lactic acid, malic acid, and acetic acid levels). Predictive functional genomic analysis of the bacterial community in all fermented soybean samples was performed using Phylogenetic Investigation of Communities by Reconstruction of Unobserved States (PICRUST) 1.0.0 based on the Greengene 16S rRNA gene dataset.

## Results

### Physicochemical Properties of Dajiang and Sufu

**FIGURE 1:**
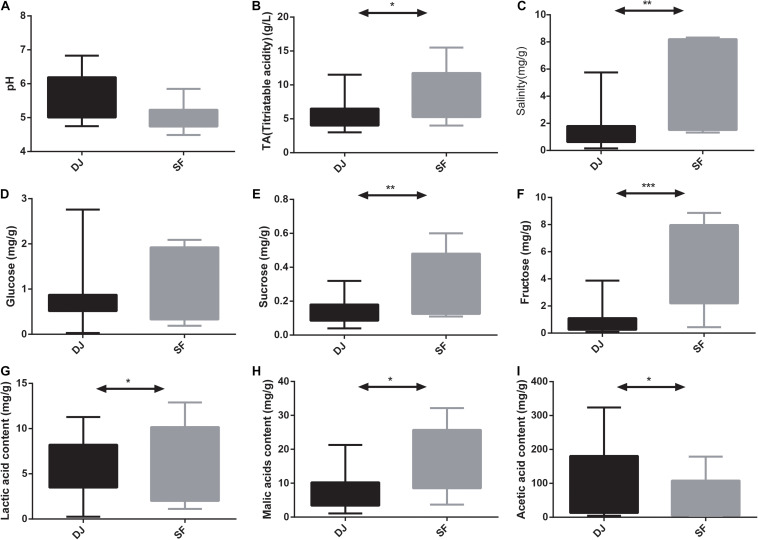
Comparison of physicochemical properties between Dajiang and Sufu. **(A)** pH; **(B)** titratable acidity (TA); **(C)** salinity; **(D)** glucose; **(E)** sucrose; **(F)** fructose; **(G)** lactic acid; **(H)** malic acid; **(I)** acetic acid. Statistical significance between groups was determined using *t*-tests. *P < 0.05, **P < 0.01, ***P < 0.001.

The pH values of Dajiang and Sufu range from 4.49 to 6.83 with no statistical differences observed between the two sample groups (Figure 1A). Meanwhile, the content of glucose was also no significant difference between the two groups (Figure 1D). However, the TA, salinity, sucrose, fructose, lactic acid, acetic acid, and malic acid levels differed significantly between Dajiang and Sufu samples (Figures 1B,C,E–I). Specifically, the Sufu samples had a higher TA, salinity, sucrose, fructose, lactic acid, and malic acid levels to the Dajiang samples (*P* < 0.05); whereas, the acetic acid level was significantly higher in Dajiang samples (*P* < 0.05).

### Comparison of Diversity Indices Between Dajiang and Sufu Samples

**FIGURE 2:**
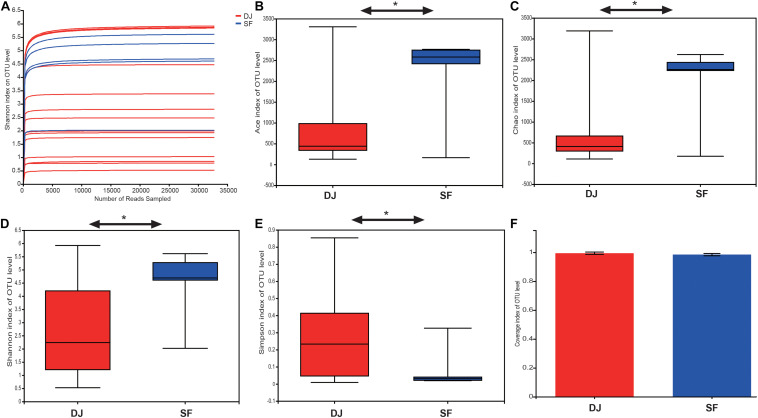
Alpha-diversity and similarity of bacterial communities in Dajiang and Sufu. **(A)** Shannon curve; **(B)** Ace; **(C)** Chao1; **(D)** Shannon index; **(E)** Simpson index; **(F)** Coverage; Statistical significance between groups was determined using *t*-tests *P < 0.05.

A total of 950,761 raw sequences with an average read length of 423.8 bp was obtained from the 19 fermented soybean samples. Details of the sequencing data and the alpha-diversity are shown in Table 1. The Shannon curves based on OTUs reached a plateau, and the coverage estimators ranged from 97.43 to 99.93, revealing that the sequencing depth was adequate for further bacterial community analysis in all samples (Figures 2A,F and Table 1). The alpha-diversity of the samples is represented by Ace, Shannon, Chao1, and Simpson indices in Figure 2. Sufu samples had the highest Ace, Shannon, and Chao1 indices compared to the Dajiang group (Figures 2B–D). Meanwhile, the values of the Simpson indices in Dajiang samples were higher than those in Sufu samples (Figure 2E).

**TABLE 1:** Sequence information and diversity values of Dadiang and Sufu in North China.

Sample_name	Seq_num	Average length (bp)	OTU	Sobs	Shannon	Simpson	Ace	Chao1	Coverage
DJ_143	70154	417.7	2374	2374	5.87	0.01	3,305.33	3,188.00	97.43
DJ_146	41444	419.9	2246	2246	5.84	0.01	2,712.70	2,553.81	98.24
DJ_50	48668	418.7	479	479	4.47	0.04	488.68	493.88	99.93
DJ_145	41509	427.5	223	223	1.93	0.27	675.77	433.52	99.66
DJ_49	58533	424.8	259	259	2.80	0.17	279.91	277.53	99.89
DJ_246	43368	428.9	79	79	0.79	0.71	160.19	108.33	99.90
DJ_247	52326	429.5	110	110	1.99	0.27	126.76	129.46	99.93
DJ_227	43638	420.4	2263	2263	5.92	0.01	2,671.93	2,565.17	98.33
DJ_241	39711	429.4	314	314	0.52	0.85	1,003.85	709.81	99.44
DJ_240	55555	428.7	166	166	2.48	0.20	369.50	292.07	99.81
DJ_391	43382	425.8	320	320	3.38	0.08	362.29	364.25	99.82
DJ_393	45792	428.5	184	184	1.74	0.30	332.68	311.11	99.79
DJ_400	39914	407.7	290	290	0.85	0.76	388.08	383.02	99.71
DJ_401	40665	428.3	212	212	1.03	0.45	917.62	511.46	99.60
SF_224	69723	422.7	1608	1608	4.61	0.04	2,417.59	2,259.50	98.07
SF_245	59811	422.7	1873	1873	5.27	0.02	2,579.58	2,429.58	98.04
SF_244	52954	423.4	1487	1487	4.69	0.03	2,764.09	2,235.41	98.14
SF_394	51566	420.7	2242	2242	5.61	0.02	2,744.76	2,619.71	98.13
SF_249	52048	427.7	131	131	2.02	0.33	164.07	173.27	99.91

**FIGURE 3:**
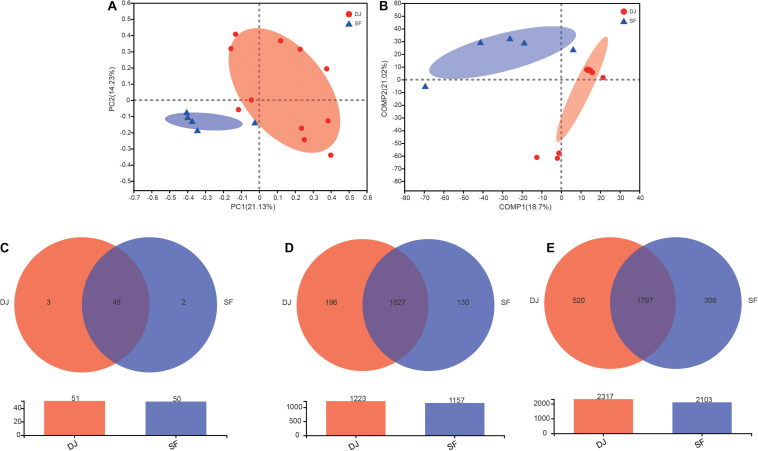
Beta-diversity and Venn diagrams of bacterial communities in Dajiang and Sufu. **(A)** Principal component analysis (PCA); **(B)** Partial least squares discriminant analysis (PLS-DA); **(C)** Venn diagrams at the phylum level; **(D)** Venn diagrams at the genus level; **(E)** Venn diagrams at the species level.

Unifrac β-diversity (unweighted) results, at the OTU level, are presented in Figure 3. Principal component analysis (PCA) revealed that the bacterial composition of Dajiang and Sufu samples differs (Figure 3A). Meanwhile, the partial least squares discriminant analysis (PLS-DA) result showed that all samples were separated into two clusters (Figure 3B), consistent with the PCA results.

The common and unique phyla, genera, and species between Dajiang and Sufu groups are depicted in a Venn diagram in Figures 3C–E. At the phylum level, 51, and 50 phyla were obtained from Dajiang and Sufu samples, respectively, of which 48 were shared between different samples (Figure 3C). At the genus level, 1027 were shared, while 196 and 130 genera were unique to Dajiang and Sufu samples, respectively (Figure 3D). At the species level, 1,797 were common between samples; while 520 species were unique to Dajiang samples and 306 were unique to Sufu (Figure 3E).

Cumulatively, these results indicate that unique bacterial communities within the Dajiang and Sufu samples from North China.

### Bacterial Profiles of Dajiang and Sufu

**FIGURE 4:**
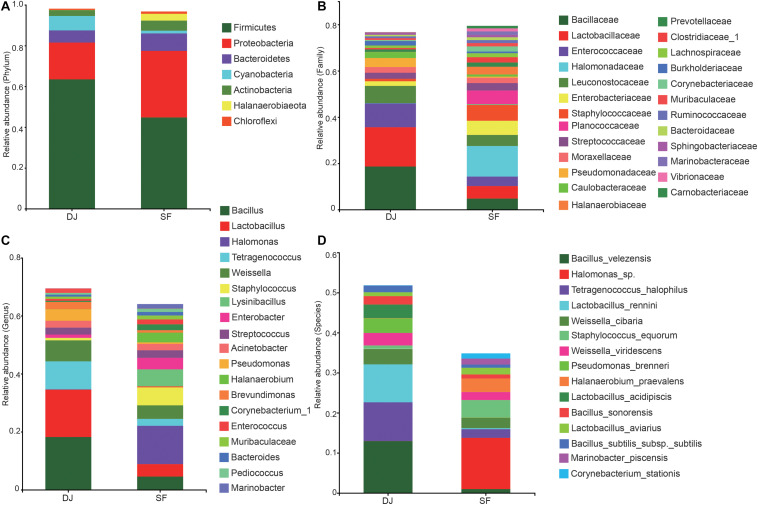
Relative abundance of the bacterial community at the phylum **(A)**, family **(B)**, genus **(C)**, species **(D)** levels in Dajiang and Sufu. Phyla, family, genus, and species with proportions less than 1% are not listed.

16S rRNA gene sequencing showed that the microbial communities of all samples included 53 phyla, 562 families, 1,353 genera, and 2,623 species. At the phylum level (Figure 4A), Firmicutes and Proteobacteria predominated in both Dajiang and Sufu samples. The relative abundance of Firmicutes in the Dajiang samples (mean average 63.5%) was higher than that in Sufu samples (mean average 44.8%). Meanwhile, the relative abundance of Proteobacteria was 18.0 and 32.7% in the Dajiang and Sufu samples, respectively. Additionally, Bacteroidetes and Actinobacteria were commonly detected in Dajiang and Sufu samples. Interestingly, these results revealed that Cyanobacteria (7.09%) were also enriched in Dajiang samples.

At the family level (Figure 4B), three major families were identified in all samples, including Bacillaceae, Lactobacillaceae, and Enterococcaceae. The relative abundance of Bacillaceae and Lactobacillaceae in Dajiang samples (18.6 and 17.0%, respectively) was higher than that in Sufu (4.8 and 5.4%, respectively) samples. Moreover, the relative abundance of Enterococcaceae in Dajiang (10.3%) samples were higher than that in Sufu (4.1%) samples. Furthermore, Dajiang samples contained Pseudomonadaceae, Streptococcaceae, Staphylococcaceae, Moraxellaceae, Caulobacteraceae, and Burkholderiaceae. Notably, other bacterial families, including Leuconostocaceae, Halomonadaceae, Enterobacteriaceae, Streptococcaceae, Staphylococcaceae, Moraxellaceae, and Planococcaceae that were observed in the Sufu samples, were absent, or scarcely detected in Dajiang samples.

At the genera level (Figure 4C), *Bacillus*, *Lactobacillus*, *Tetragenococcus*, and *Weissella* were the most abundant in the two groups. *Bacillus* and *Lactobacillus* were the predominant genera in Dajiang samples with relative abundances much higher than those in Sufu groups. The other highly abundant genera in Dajiang samples included *Pseudomonas* (3.90%), *Streptococcus* (2.39%), *Acinetobacter* (2.37%), *Enterobacter* (1.12%), *Staphylococcus* (1.06%), and *Brevundimonas* (2.50%), respectively. Meanwhile, the bacterial composition of Sufu samples was more complex. Aside from *Bacillus*, *Lactobacillus*, and *Tetragenococcus*, *Halomonas* was the most predominant genera in Sufu samples, accounting for 13.2%. Furthermore, the relative abundances of *Streptococcus*, *Acinetobacter*, *Staphylococcus*, *Enterobacter*, *Lysinibacillus*, *Halanaerobium*, *Enterococcus*, *Corynebacterium_1*, *Bacteroides*, *Pediococcus*, *Sphingobacterium*, and *Marinobacter* were enriched in Sufu samples.

At the species level (Figure 4D), *Bacillus velezensis* (12.95%), *Tetragenococcus halophilus* (9.66%), and *Lactobacillus rennini* (9.48%) were the most abundant species in the Dajiang groups. However, these samples also contained high relative abundance of *Weissella cibaria*, *Weissella viridescens*, *Pseudomonas brenneri*, and *Lactobacillus acidipiscis*. Interestingly, *Halomonas* sp., and *Halanaerobium praevalens* were enriched in the Sufu samples, while being absent or scarcely detected in Dajiang samples.

### Differential Bacteria of Dajiang and Sufu

**FIGURE 5:**
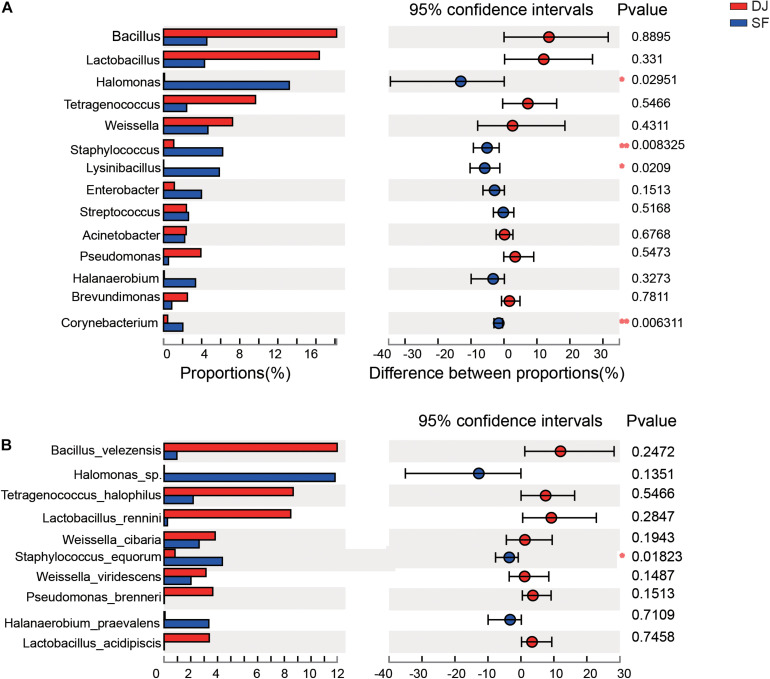
Linear discriminant analysis effect size of Dajiang and Sufu. **(A)** Histogram of the microbiota at the genus level. **(B)** Histogram of the microbiota at the species level. *P < 0.05, **P < 0.01.

**FIGURE 6:**
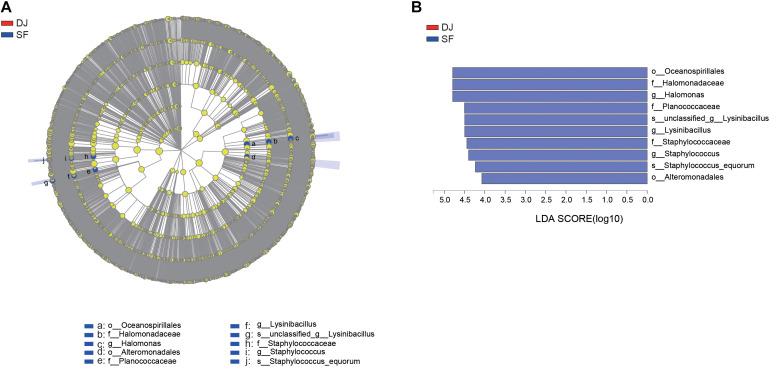
LEfSe comparison of bacterial communities in Dajiang and Sufu. **(A)** Cladogram representing the abundance of bacterial taxa in Dajiang and Sufu. **(B)** Histogram depicting the microbiota in Dajiang and Sufu with a threshold value of 4.

According to the relative abundance of the bacterial organisms, an analysis of microbial communities in the different groups was carried out using the Kruskal–Wallis H test (Figure 5) and Linear Discriminant Analysis Effect Size (LEfSe) analysis (Figure 6). At the genus level, the relative abundance of *Halomonas*, *Staphylococcus*, and *Lysinibacillus* in Sufu samples were significantly higher than Dajiang samples (*P* < 0.05); while at the species level, the *Staphylococcus equorum* population in Sufu samples was higher than in Dajiang samples (*P* < 0.05; Figure 5). These results were consistent with those of LEfSe with three genera, namely *Halomonas*, *Staphylococcus*, and *Lysinibacillus*, enriched in Sufu samples compared to Dajiang samples, and one species (*S. equorum*) differentially abundant in Sufu samples (Figure 6).

### Correlation Analyses Between Bacteria and Physicochemical Indices of Dajiang and Sufu

**FIGURE 7:**
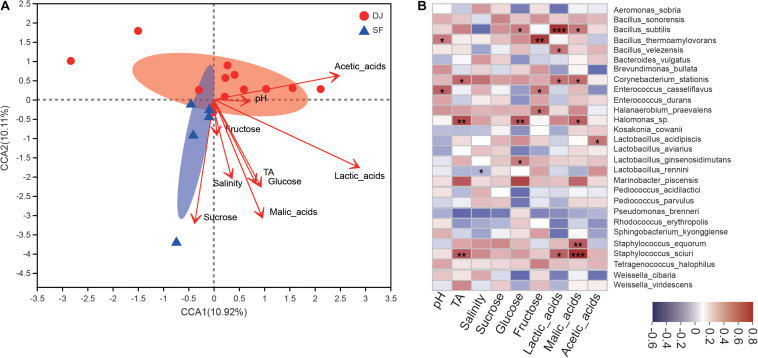
Correlational analyses. **(A)** Canonical correlation analysis (CCA) of the Dajiang and Sufu bacterial communities. **(B)** Heatmap of Spearman correlations between microbiota and physicochemical characteristics at the species level in different fermented soybean products. R values represent positive (red) or negative (blue) correlations. *P < 0.05, **P < 0.01, ***P < 0.001.

Correlations between microbial communities and physicochemical properties have been analysed using Spearman correlation analysis and are presented in Figure 7.

The results of canonical correlation analysis (CCA) analysis (Figure 7A) suggest a strong correlation between physicochemical indices and bacterial composition. Lactic acid had the greatest effect on bacterial composition, followed by malic acid. Meanwhile, pH, TA, salinity, sucrose, glucose, fructose, and acetic acid did not influence the bacterial composition. According to the Mantel test, lactic acid (*r* = 0.4081, *P* = 0.013) had the strongest correlation with microbial abundance, followed by malic acid (*r* = 0.3515, *P* = 0.043). Additionally, sucrose contents (*r* = 0.3559, *P* = 0.056) were weakly correlated with microbial composition. Furthermore, *Bacillus subtilis* was more strongly correlated with lactic acid levels, *L. acidipiscis* was positively correlated with acetic acid levels, while *Staphylococcus sciuri* and *S. equorum* were more strongly correlated with malic acids (Figure 7B).

### Predicted Functions of the Bacterial Communities in Dajiang and Sufu Samples

**FIGURE 8:**
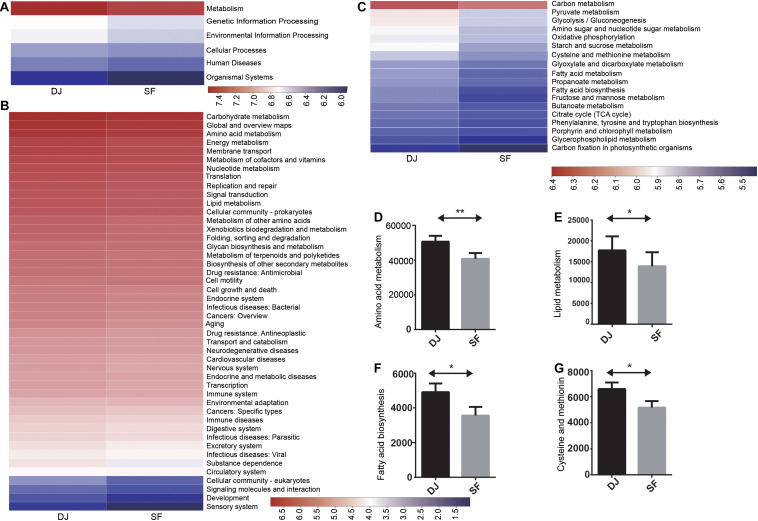
Predictive functions of the bacterial communities in Dajiang and Sufu products. **(A)** Overall Kyoto Encyclopedia of Genes and Genomes (KEGG) gene function statistics (Level 1). **(B)** Heatmap of functional pathways (Level 2). **(C)** Heatmap of functional pathways (Level 3). **(D)** Amino acid metabolism. **(E)** Lipid metabolism. **(F)** Fatty acid biosynthesis. **(G)** Cysteine and methionine metabolism. *P < 0.05, **P < 0.01.

The biological functions of microbial genes identified in all samples were predicted by PICRUST. Additionally, Kyoto Encyclopedia of Genes and Genomes (KEGG) pathway analysis provided a better understanding of the functional genes associated with the different microbial communities. The identified bacterial genes were found to be enriched in pathways associated with metabolism, genetic information processing, environmental information processing, cellular processes, human diseases, and organismal systems (Figure 8A). Metabolism-related genes were the most abundant, with no significant difference detected between the Dajiang and Sufu samples, suggesting that metabolism served an important role in the bacterial communities of all tested samples. Meanwhile, analysis of level-2 KEGG functional metabolism-related genes revealed that carbohydrate metabolism was the largest pathway in these two groups (Figure 8B). In addition, energy metabolism, metabolism of cofactors and vitamins, and nucleotide metabolism were also abundant in both groups. However, the Dajiang group exhibited higher amino acid metabolism (*P* < 0.05) and lipid metabolism (*P* < 0.05) compared to the Sufu group. Notably, membrane transport genes were higher in all samples, indicating its key role in these bacteria. However, a few genes related to human diseases were also detected, implying that pathogenic bacteria might have entered the Dajiang and Sufu fermentation products. Thus, the pathogenicity of these bacteria requires further investigation. At level 3, the pathways of pyruvate metabolism, glycolysis/gluconeogenesis, amino and nucleotide sugar metabolism, starch and sucrose metabolism, as well as glyoxylate and dicarboxylate metabolism, were enriched in all samples (Figure 8C). Additionally, glycine, serine, and threonine metabolism, as well as that of alanine, aspartate, and glutamate were enriched in all samples. Moreover, fatty acid biosynthesis, as well as cysteine and methionine metabolism were higher in the Dajiang samples compared to the Sufu samples (*P* < 0.05).

## Discussion

In this study, the physicochemical indices, and bacterial profiles of fermented soybean products (Dajiang and Sufu) were characterised and compared. Furthermore, the functions of microbiota in the Dajiang and Sufu groups were elucidated.

TA, pH, and salinity are three basic indexes of fermented foods that not only influence microbial structure, but also determine the quality and storability of fermented foods (Lee et al., 2017). Overall, the TA and salinity contents in the Sufu samples were significantly higher than those in the Dajiang group. During the soybean fermentation process to product Dajiang and Sufu, the bacterial community utilises carbon and nitrogen sources, resulting in the production of different metabolites, including organic acids and isoflavones, which contribute to the characteristic taste, flavour, and quality of the final product (Mouritsen et al., 2017). Overall, the Sufu samples had higher lactic acid and malic acid content compared to the Dajiang samples, indicating that there are major differences between Dajiang and Sufu in their physical and chemical properties, which may be due to the differences in microbial composition during the fermentation process (Cao et al., 2017).

The Ace and Chao1 alpha-diversity indices demonstrate bacterial richness, while the Shannon and Simpson index represent bacterial community diversity (Cao et al., 2017). Although Sufu samples harboured much higher bacterial diversity than did Dajiang samples, Firmicutes and Proteobacteria were the two predominant phyla in both samples, consistent with previous studies in fermented soybean products (Wan et al., 2020; Xie et al., 2020). Meanwhile, *Bacillus*, *Lactobacillus*, and *Tetragenococcus* were the most common abundant genera in all samples, which agrees with the results of previous studies in soybean paste samples collected from different countries (Sun et al., 2018). *Bacillus* produces potential enzymes, such as protease (Pant et al., 2015; Chen Y. H. et al., 2018), amylase (Satomi et al., 2014), and lipase (Ma et al., 2018) that are predominant in other fermented soybean products, such as doenjang-meju and Sufu. *Lactobacillus*, a major lactic acid bacterial genus, utilises carbohydrates to produce lactic acid and certain fatty acids (Gnzle, 2015; Xiao et al., 2018), which inhibit the growth of pathogenic microorganisms. *Lactobacillus* also degrades arginine, histidine, and aspartic acid, contributing to the flavour of soybean pastes (Xie et al., 2019). Moreover, some *Tetragenococcus* spp., a salt-tolerant lactic acid bacteria, counteract *Aspergillus oryzae* in soy sauce brewing (Ikuko et al., 2018). Therefore, *Bacillus*, *Lactobacillus*, and *Tetragenococcus* may be inoculated to improve fermentation efficiency and quality of Dajiang and Sufu through multi-strain culture fermentation.

This microbial community structure analysis revealed that the predominant bacterial communities within the Dajiang and Sufu groups differed greatly. Specifically, *B. velezensis*, *T. halophilus*, and *L. rennini* were more abundant in Dajiang samples. Similarly, *B. velezensis* SSH100-10 has been isolated from Korean traditional soy sauce (Chang et al., 2012). Meanwhile, *T. halophilus* may be important for the production of organic acids, amino acids, and flavouring compounds during fermentation of salty foods (Jong-Hoon et al., 2017). Additionally, *T. halophilus* has health benefits, including immunomodulatory properties and amelioration of atopic diseases (Ohata et al., 2011). It is, therefore, recommended to use *T. halophilus* as a starter culture to improve the flavour, taste, and quality of fermented soybean products (Chun et al., 2019). *L. rennini*, produces γ-aminobutyric acid (GABA), even with coexisting lactic acid and common salt in the medium (Hanya et al., 2012). GABA has essential roles in cardiovascular and cerebral blood pressure, promotes the balance of amino acid metabolism in human health, and regulates immune function (Mirzaei-Damabi et al., 2020). In addition, the results indicated that *P. brenneri* was also dominant in Dajiang samples. In fact, Yazourh et al. reported that *P. brenneri* has been isolated from natural mineral waters and raw milk (Yazourh et al., 1996). They also indicated that it utilises glucose, trehalose, 2-ketogluconate, inositol, L-valine, and γ-alanine, while producing fluorescent pigments, catalase, cytochrome oxidase, and lecithinase. The presence of higher abundance of *P. brenneri* in Dajiang samples might be due to the production environments, such as differences in air, water, and fermentation equipment. *W. cibaria* and *L. acidipiscis* were also commonly detected in Dajiang samples. Meanwhile *Halomonas*, *Lysinibacillus*, and *Staphylococcus* spp. predominated in Sufu samples, however, were not detected, or detected at very low levels, in Dajiang samples. These results differ slightly from previous studies, which reported the dominant bacterial species in Sufu samples as *Brevibacterium* (Xie et al., 2018). Regardless of these slight differences, the results of the previous study, as well as those of the current study, clearly indicate that the bacterial community in Sufu samples is distinct from that of Dajiang.

Current research has indicated that there may be safety risks associated with microbial contamination in traditional fermented foods (Cao et al., 2017; Liu et al., 2019). *Staphylococcus*, an opportunistic pathogen, which reportedly causes septicaemia, endocarditis, pneumonia, and meningitis by producing a variety of exotoxins and enzymes (Daniel et al., 2014), was observed to be more abundant in the Sufu samples. This may be due to the specific production processes associated with Sufu; that is, *Staphylococcus* organisms may contaminate the fermentation product from the external environment. Meanwhile, *W. viridescens*, a heat-resistant strain, was enriched in Dajiang samples; this species has also been isolated from perishable cooked meat products and has been described as a cause of spoilage in these products (Kameník et al., 2015). Thus, it is crucial to take appropriate measures to inhibit potentially pathogenic bacteria and other bacterial contaminants in fermented soybeans.

As mentioned above, the results of the current study cumulatively imply that the differences in the bacterial composition might be caused by differing starting raw materials and fermentation conditions, including the specific facility used for the fermentation process, as well as the applied temperature and duration. Specifically, the fermentation time required for Sufu is generally 20–30 days, while that for Dajiang is approximately 3–4 months. We, therefore, postulate that the elevated concentration of acetic acid in Dajiang, compared to Sufu samples, may have been caused by its accumulation over the extended fermentation period. Consequently, the different bacterial communities may arise due to the varying acid contents between Dajiang and Sufu.

Statistical analyses revealed a moderate positive correlation between the bacterial communities and physicochemical characteristics of Dajiang and Sufu. Specifically, pH, salinity, and TA were significantly correlated with many important species, suggesting that these physicochemical properties could be used as quality indicators during the Dajiang and Sufu fermentation process. A previous study suggested that glucose, sucrose, and fructose are important carbon sources for the metabolic activities of microorganisms (Jung et al., 2014). Meanwhile, the current study results show that glucose is significantly correlated with *B. subtilis*, *Halomonas* sp., and *Lactobacillus ginsenosidimutans*, while fructose is correlated with *Bacillus thermoamylovorans*, *Enterococcus casseliflavus*, and *H. praevalens*. Moreover, bacterial metabolites, including organic acids such as lactic acid and acetic acid, represent important factors in the generation of the characteristic flavour, odour, and taste of fermented soybean products (Datta and Henry, 2010). In this study, acetic acid levels were positively correlated with *L. acidipiscis*, malic acid levels were positively correlated with *Halomonas* sp., and lactic acid levels were significantly correlated with *B. subtilis* and *B. velezensis*, but not with *Lactobacillus*. These results are inconsistent with previous studies (Rui et al., 2017; Xiao et al., 2018), suggesting that *Lactobacillus* is positively correlated with lactate in Sichuan Paocai. Based on these results, it was concluded that *Lactobacillus*, *Bacillus*, and *Halomonas*, which have a strong correlation with organic acids, also play important roles in the fermentation of Dajiang and Sufu. In addition, these results indicate that *Lactobacillus* and *Bacillus* are abundant in traditional Dajiang samples, while *Halomonas* is enriched in Sufu samples, which might explain the higher acetic acid content in Dajiang and the higher malic acid content in Sufu samples.

Additionally, the functions associated with the unique bacterial communities in both groups were also investigated. Results showed that carbohydrate metabolism-related genes were the most abundant in all groups, suggesting that vigorous carbohydrate metabolism occurred in all samples. Moreover, amino acid metabolism and lipid metabolism-related genes were more abundant in Dajiang samples compared to Sufu (Figures 8D,E). Amino acid metabolism may be involved in the production of small amino acids or peptides, which may act as flavour substances (Collar et al., 2010). Lipid metabolism may also contribute to the production of fatty acids with different chain lengths (Gaenzle et al., 2007). These results suggest that the observed differences might contribute to the unique flavours of Dajiang and Sufu, which required further investigation in subsequent studies. Moreover, the relatively higher level of cysteine and methionine metabolism, and fatty acid biosynthesis-related genes in Dajiang samples compared to Sufu (Figures 8F,G), was consistent with the higher acetic acid content.

Certain limitations were noted in this study. First, only a few Sufu samples were included in the analysis, which may have affected the experimental results. Thus, the study should be repeated with a larger number of samples. Second, previous studies have reported that the presence of fungi, especially yeast, has a significant impact on soybean flavour and taste (Wan et al., 2020), thus further studies are required to also assess the fungal species via internal transcribed spacer and 18S rRNA high-throughput sequencing.

## Conclusion

In summary, the physicochemical indicators, and microbial communities in traditional Dajiang and Sufu collected from North China were characterised and compared. Significantly higher amounts of TA, salinity, sucrose, fructose, lactic acid and malic acid were found in Sufu samples, while higher contents of acetic acid were observed in Dajiang samples. In addition, four genera (*Halomonas*, *Staphylococcus*, *Lysinibacillus*, and *Corynebacterium*) and one species (*S. equorum*) were enriched in Sufu samples. Meanwhile, acetic acid and sucrose proved to be the primary physicochemical factors influencing the bacterial communities in Dajiang and Sufu, respectively. Finally, genes related to carbohydrate metabolism were enriched in Dajiang, particularly those associated with amino acid metabolism, cysteine and methionine metabolism, as well as those related to fatty acid biosynthesis. Taken together, these results provide a basic understanding of the bacterial communities in Dajiang and Sufu, and may prove useful for the identification of functional bacteria suitable for Dajiang and Sufu production. However, the key bacterial strains responsible for the development of organic acids in these two food products must be identified, while their specific functions should be investigated using metagenomics, metaproteomics, and meta-transcriptomics *in vitro* and *in vivo* studies.

## Data Availability Statement

The original contributions presented in the study are included in the article/supplementary material, further inquiries can be directed to the corresponding authors.

## Author Contributions

TL, XX, XC, JZ, YD, and QW conceived and designed the experiments, authored or reviewed drafts of the manuscript, and approved the final draft. JM, LW, YX, HZ, LL, HL, YF, JZ, LX, and MC collected the samples, performed the experiments, analysed the data, and prepared figures and tables. All authors contributed to the article and approved the submitted version.

## Conflict of Interest

The authors declare that the research was conducted in the absence of any commercial or financial relationships that could be construed as a potential conflict of interest.

## Publisher’s Note

All claims expressed in this article are solely those of the authors and do not necessarily represent those of their affiliated organizations, or those of the publisher, the editors and the reviewers. Any product that may be evaluated in this article, or claim that may be made by its manufacturer, is not guaranteed or endorsed by the publisher.

## Footnotes

1
http://www.mothur.org

2
http://cran.r-project.org/
